# Comment/response to letter to editor

**DOI:** 10.1080/2090598X.2020.1846280

**Published:** 2020-11-09

**Authors:** Vinita Agrawal, Niharika Bharti, Rakesh Pandey

**Affiliations:** Department of Pathology, Sanjay Gandhi Postgraduate Institute of Medical Sciences, Lucknow, India, vinita.agrawal15@gmail.com

We thank the authors for the letter to Editor. In response, we would like to clarify that one-third of the high-grade non-invasive bladder urothelial carcinomas showed HER2 3+ protein expression on immunohistochemistry. About one-half of these HER2 3+ tumours show HER2 gene amplification and can benefit from HER2-targeted therapy.

